# Incidence, prevalence and characteristics of multimorbidity in different age groups among urban hospitalized patients in China

**DOI:** 10.1038/s41598-023-46227-4

**Published:** 2023-11-01

**Authors:** Dixiang Song, Deshan Liu, Weihai Ning, Yujia Chen, Jingjing Yang, Chao Zhao, Hongwei Zhang

**Affiliations:** Department of Neurosurgery, Sanbo Brain Hospital, Capital Medical University, Beijing, China

**Keywords:** Geriatrics, Public health, Health care economics

## Abstract

The aim of the study was to investigate the incidence, prevalence and characteristics of multimorbidity in urban inpatients of different age groups. This study used data from the National Insurance Claim for Epidemiology Research (NICER) to calculate the overall incidence, prevalence, geographic and age distribution patterns, health care burden, and multimorbidity patterns for multimorbidity in 2017. According to our study, the overall prevalence of multimorbidity was 6.68%, and the overall prevalence was 14.87% in 2017. The prevalence of multimorbidity increases with age. The pattern of the geographic distribution of multimorbidity shows that the prevalence of multimorbidity is relatively high in South East China. The average annual health care expenditure of patients with multimorbidity increased with age and rose rapidly, especially among older patients. Patients with cancer and chronic kidney disease have higher treatment costs. Patients with hypertension or ischemic heart disease had a significantly higher relative risk of multimorbidity than other included noncommunicable diseases (NCDs). Hyperlipidemia has generated the highest number of association rules, which may suggest that hyperlipidemia may be both a risk factor for other NCDs and an outcome of them.

## Introduction

Noncommunicable diseases (NCDs), also known as chronic diseases, tend to be of longer duration and are the result of a combination of genetic, physiological, environmental and behavioral factors. The main types of NCDs are cardiovascular diseases (e.g., heart attacks and stroke), cancers, chronic respiratory diseases (e.g., chronic obstructive pulmonary disease and asthma) and diabetes. Cardiovascular disease is the most prominent NCD causing premature death^1^. NCDs caused 65% of all-cause deaths in 2010^2,3^. The term ‘‘multimorbidity’’ is often defined as the cooccurrence of multiple chronic conditions within the same individual^4^. In people with multimorbidity, on the one hand, the results in higher mortality and disability rates, higher risk of adverse drug events, lower functional status and lower quality of life than having only one NCD^5^. On the other hand, the corresponding medical costs of a multimorbidity state can increase considerably, rather than simply doubling, in turn place an enormous burden on the patient's family and national health care expenditure^6,7^.

The incidence of multimorbidity in China increases with age, and the pattern of multimorbidity varies somewhat by age group^8–10^. It is therefore important to improve the understanding of the treatment of multimorbidity and to study the incidence, prevalence and characteristics of multimorbidity in China, especially in different age groups. There have been numerous previous epidemiological studies of multimorbidity based on Chinese community or inpatient populations, but the findings vary widely among them. These studies have several limitations, such as relatively limited sample sizes and incomplete coverage of the sample population. For example, Cheng et al.^9^, Yao et al.^8^, Zhao et al.^6^, Chen et al.^11^ used data from the China Health and Retirement Longitudinal Study (CHARLS), adopting a sample of less than 20,000 people from 150 counties in 28 provinces, giving a broad but relatively small sample size of the population. Zhang et al. conducted a multicenter, multimorbidity study of 4633 inpatients aged 60 years or older recruited from 12 hospitals in 7 cities in China through the China Comprehensive Geriatric Assessment Study conducted in 2011–2012^12^. The coverage and sample size of the population sample are limited. Zhang et al. used data from the Beijing Longitudinal Study of Aging (BLSA) to study the prevalence and trends of multimorbidity in a community of 6593 Beijing residents aged 60 years and older, focusing on development over time scales of time^13^. Wang, Xiaowen et al. included 2.1 million middle-aged adults aged > 45 years in a chronic disease study using the Beijing Medical Claim Data for Employees (BMCED) from 2011 to 2015^14^. Zhang, Jiao et al. included 5493 elderly people aged 65 years or older from the eastern coast of Shandong to study the prevalence of obesity and multimorbidity. These three studies were all conducted only in a particular provincial area or special administrative region. First, most of the studies were based on small surveys with a limited population distribution^15^. China is a vast, geographically and climatically diverse country, and the pattern of multimorbidity varies from one area to another^6,8,11,13,16^. Thus, it is difficult to obtain a good representation of these studies. Second, most epidemiological studies of NCDs or multimorbidity have used strategies that predefine the study disease, and the different definitions have largely influenced the results. In addition, the method of data collection (e.g., self-administered questionnaire interviews, social worker evaluation questionnaires, medical records), the age range of the respondents and the source of the data also had an influence on the reporting rate of diseases^17^.

In addition, in the management of patients with multiple diseases, we are often confused by conflicting recommendations given by different disease guidelines. Therefore, it is important for us to understand more about multimorbidity patterns to provide better judgment and more comprehensive treatment and health advice to these patients^18–21^. Some of the patterns of multimorbidity identified in previous studies include cardiovascular and metabolic diseases (mainly diabetes, hypertension, heart disease, hyperlipidemia and obesity), mental health problems (mainly depression and anxiety) and musculoskeletal disorders (arthropathy, back/neck pain, and osteoporosis)^18^. Among elderly individuals, there are mainly cardiometabolic (CM), mechanical (MEC) and psychogeriatric (PG) conditions^22^. However, the only one that is more accepted is cardiovascular and metabolic diseases^18^. This pattern of multimorbidity may describe the same phenomenon as metabolic syndrome. Beyond this, more research may be needed to confirm the multimorbidity pattern.

We aim to explore the patterns and prevention priorities of multimorbidity through a large sample size and wide coverage of data with the objectives of incidence, prevalence and prevalence patterns of NCDs and multimorbidity with targets such as regional and demographic subgroups to better understand the characteristics of multimorbidity in urban China.

## Materials and methods

### Data source

This study is from the National Insurance Claim for Epidemiology Research (NICER) database of the China Health Insurance Database, a study based on statistical-level data from a dynamic cohort of basic health insurance. The database covers health insurance information for outpatients and inpatients in all 31 provinces, including statistical information on enrollment, diagnosis of diseases, and billing of medical expenses. The enrollment and billing statistical information tables are stored separately. The geographical dimension of the statistics is coordinated by municipality in each region and in a few regions by province or county and is updated monthly. In most regions, the data have been entered into the database since January 2012. By the end of 2017, 295 million urban workers and 448 million urban residents were insured in China's health insurance database. Several previous studies based on this database have been conducted^23–31^.

Informed: the study has obtained data use consent forms and confidentiality agreements with the data managers. All data used have been deidentified and are exclusively for the purposes of this research. The authors did not have access to information that could identified individual participants during or after data collection. The data in this study do not involve any human experimentation or the use of human tissue samples. All methods were carried out in compliance with relevant guidelines and regulations. The Institutional Review Board of Capital Medical University Sanbo Brain Hospital approved the data analysis and waived the requirement for informed patient consent.

### Study population

Patients whose information was registered in the NICER database by UEBMI and who had an outpatient (including emergency) or inpatient visit between 2017.1.1 and 2017.12.31 were included in this study.

Inclusion criteria:

1. age ≥ 18 years;
2. outpatient diagnosis missing < 20%;
3. Patients with complete diagnostic information including primary and secondary diagnoses;
4. at least one diagnosis recorded during 2017.

Exclusion criteria:

1. total missing diagnoses > 1%;
2. key variables missing. The sampling method was performed via stratified sampling.

### Definition and identification of diagnosis

In this study, the term NCDs adheres to the World Health Organization's definition, characterizing them as a group of conditions primarily independent of acute infections, resulting in persistent health implications and often requiring long-term treatment and care. We defined 12 common NCDs based on reports from the Chinese Health Statistical Yearbook and the incidence and prevalence of NCDs/multimorbidity reported in the previous literature. Multimorbidity was identified when two or more NCDs as defined above were recorded during the study period.

**Figure 1:**
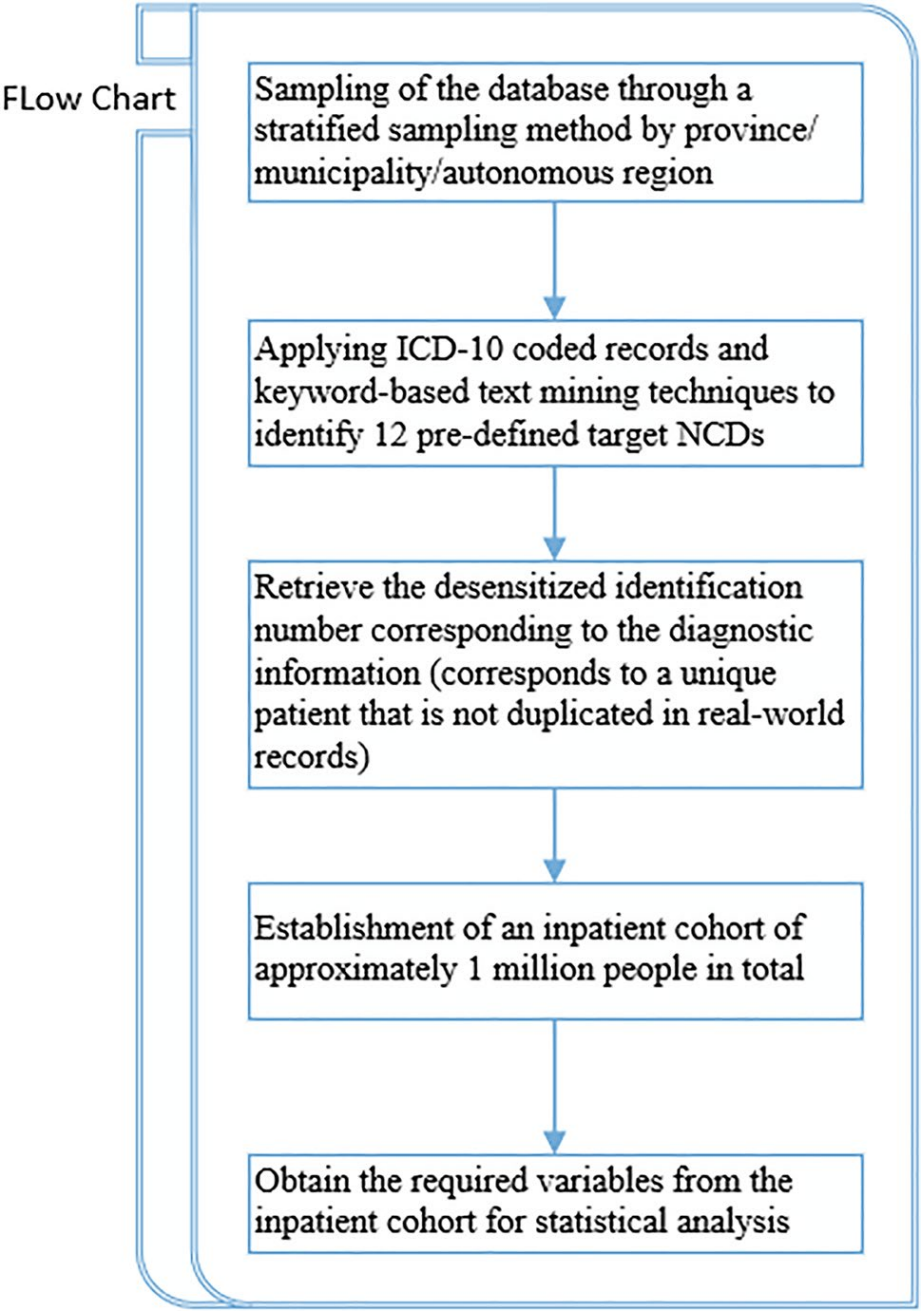
Flowchart for acquiring the study cohort. *NCDs* noncommunicable diseases.

In the database, disease diagnosis information is identified by 6 relevant variables, including 3 diagnosis name variables (e.g., primary diagnosis name, secondary diagnosis name 1, secondary diagnosis name 2) and 3 diagnosis code variables (e.g., primary diagnosis code, secondary diagnosis code 1, secondary diagnosis code 2). As only a few regional health facilities use the standardized ICD-9 or ICD-10 diagnosis codes, the ICD-10 code for the corresponding disease is only used as an aid to judgment. As long as one of the primary or secondary diagnoses can be identified as the target disease, it is counted as an individual case. A test cohort of approximately 100,000 samples was drawn, and incorrect keyword terms were extracted by and manually reviewing the top 30% of diagnostic terms accessed to exclude common mis-screened diagnoses. Figure 1 illustrates the process of obtaining the study cohort for this study.

During the study period, individuals who were identified in their medical records as having two or more distinct NCDs were categorized as having multimorbidity.

### Statistical analysis

The statistical analysis was performed using IBM SPSS Statistics 24, Python 3.9.7 and R studio 2022.07.2-576. Subjects with missing information were excluded from the relevant portion of the analysis. For descriptive statistics, quantitative information was expressed as the mean ± standard deviation for normal distributions and median and interquartile spacing for skewed distributions; qualitative information was expressed as ratio, composition ratio and rate. Analysis of the effect of age on multimorbidity was performed using binary logistic regression, with the OR and its 95% CI calculated, and p < 0.05 was considered statistically significant.

The previous 4 years of the study period (2013–2016) were set as the washout period for the 2017 inpatient population. After completing the initial data sampling, the incidence and prevalence of each NCD and multimorbidity status were calculated separately between the different age subgroups. For the calculation of the prevalence rate, the denominator consisted of the total number of subjects with continuous medical records in 2017. The numerator includes subjects diagnosed with specific NCDs or categorized as having multimorbidity between 2013 and 2017 who were also alive and had at least one medical record in 2017. For the calculation of the incidence rate, the denominator comprises the total number of subjects with continuous medical records in 2017. The numerator consists of subjects diagnosed with specific NCDs or categorized as having multimorbidity in 2017 but who were not diagnosed with the corresponding specific NCD or defined as having multimorbidity during the washout period.

To explore the relationship between age and the occurrence of multimorbidity, binary logistic regression analysis was used to calculate odds ratios (ORs) for univariate analysis.

Calculation of RR values for the occurrence of multimorbidity for each NCD:

- $$RR\, for\,each\, NCD=\frac{\text{Prevalence of multimorbidity in patients with a particular NCD}}{\text{Prevalence of multimorbidity in patients with a chronic disease other than a particular NCD}}$$
- $$RR\, for \,population\, characteristics=\frac{\text{Prevalence of multimorbidity in a characteristic population}}{\text{Prevalence of multimorbidity in populations without this characteristic}}$$

The multimorbidity pattern is calculated using a 2-step approach^32^. The clustering analysis explores the aggregation of 12 NCDs by means of the k-nearest neighbors algorithm, the Pearson correlation method as a similarity measure, and the seaborn package for Python as a clustering analysis. Furthermore, we conducted direct ARM for the 12 NCDs without employing cluster analysis. An association rule analysis (ARM) was then conducted on the diseases in the cluster to explore support (the frequency of disease combinations in the dataset), confidence (the conditional probability that a participant with an antecedent disease also has a consequent disease) and lift (the frequency of disease combinations in the dataset). The basic rule of ARM is as follows: if there is an antecedent disease, then there is a probability of a consequent disease. Support indicates how frequently the if/then relationship appears in the database, i.e., Support (A → B) = P(A ∪ B); Confidence tells about the number of times these relationships have been found to be true, i.e., Confidence (A → B) = P(B|A). When the antecedent disease x and consequent disease y are independent of each other, P(x, y) = P(x)P(y) = 1. However, if x is related to y, then the above equation does not hold; at this point, Lift can reflect the ratio of the frequency of simultaneous occurrences of the items we observed in the study to the expected frequency, i.e., $\text{Lift}=\frac{\text{ P}(\text{x},\text{y})}{\text{P}(\text{x})\text{P}(\text{y})}$. If this ratio is greater, it means that in the subset containing x, the lift in the occurrence of y is greater compared to the full set. ARM was performed using the arules package in R.

Statistical descriptive analysis encompasses population characteristics, including year of visit, brief age stratification, extended age stratification, single NCD, multimorbidity, number of chronic conditions, and multimorbidity combinations within the 2017 hospitalization cohort. The analysis of multimorbidity combinations is conducted at three levels of multimorbidity (i.e., pairs, triads, quartets and beyond). Statistical calculations are not duplicated between each level (For instance, in the count of "hypertension, diabetes," cases including "hypertension, diabetes and hyperlipidemia" are not counted). The impact of multimorbidity on the number of outpatient visits, total outpatient expenditure, number of inpatient visits, total inpatient expenditure and overall expenditure was analyzed separately through multivariate analysis. The exchange rate of RMB to USD is based on the average of 2017 exchange rates.

### Ethics approval

The study has signed data use consent forms and confidentiality agreements with the data managers, and all data used are desensitized and used only for the purposes of this research.

## Results

### Prevalence, incidence, and patterns of NCDs and multimorbidity in the overall data

To ensure data quality, we excluded 6 provinces, municipalities or autonomous regions from mainland China that had high rates of missing data, including Beijing, Fujian, Guizhou, Liaoning, Shanghai and Tibet. A total of approximately 23.02 million people in NICER had a medical visit recorded in 2017. A total of 955,354 patients with a visit recorded in 2017 were included in this study after a stratified sample was performed. Of these, 51.7% were males and 48.3% were females. The mean age was 57.81 (± 16.14) years, with 60.17 (± 15.60) years for males and 55.29 (± 16.33) years for females.

**Figure 2:**
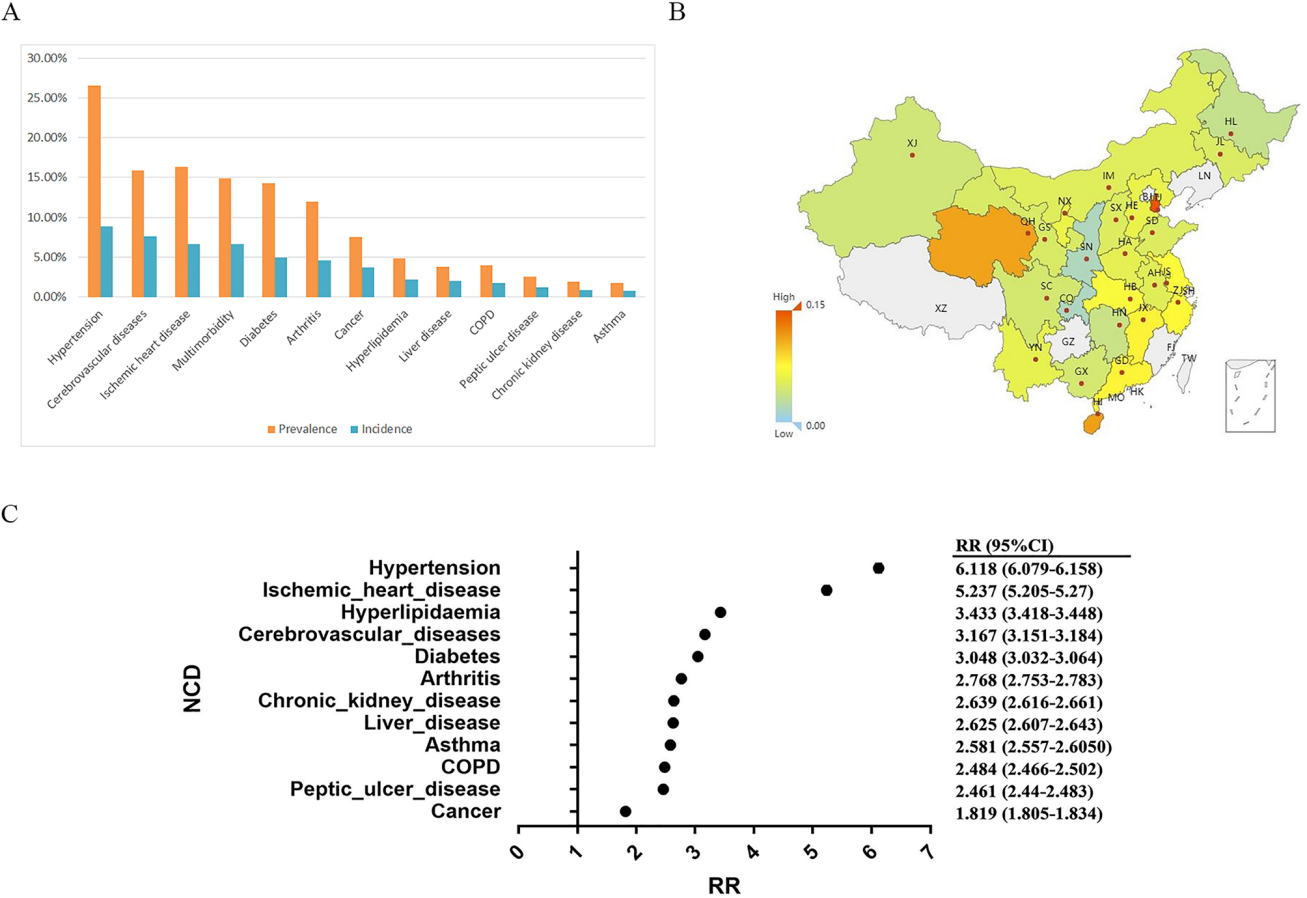
Descriptive and statistical results of multimorbidity in the study cohort. (**A**) Incidence of the 12 included NCDs in the overall population and among elderly inpatients. (**B**) Map of the incidence of multimorbidity. (**C**) Forest plots of RRs and corresponding 95% CIs for the occurrence of multimorbidity for each NCD for patients with at least one NCD (the 95% CI for RR are too narrow to be properly displayed as error bars in the forest graph). *NCD* noncommunicable disease, *COPD* chronic obstructive pulmonary disease, *AH* Anhui, *CQ* Chongqing, *GD* Guangdong, *GS* Gansu, *GX* Guangxi, *HA* Henan, *HB* Hubei, *HE* Hebei, *HI* Hainan, *HL* Heilongjiang, *HN* Hunan, *IM* Inner Mongolia, *JL* Jilin, *JS* Jiangsu, *JX* Jiangxi, *NX* Ningxia, *QH* Qinghai, *SC* Sichuan, *SD* Shandong, *SN* Shaanxi, *SX* Shanxi, *TJ* Tianjing, *XJ* Xinjiang, *YN* Yunnan, *ZJ* Zhejiang.

The incidence and prevalence of NCDs in the overall population are shown in Table 1, with the corresponding bar chart displayed in Fig. 2A. In the overall sampling population, the incidence of hypertension (HT), cerebrovascular diseases (CbVD) and ischemic heart disease (IHD) (6.73%, 5.79%, 5.08%) were the highest among the 12 NCDs we included.

**Table 1:** Incidence and prevalence of 12 included NCDs.

	Total		18–34		35–64		Age ≥ 65	
	Incidence (%)	Prevalence (%)	Incidence (%)	Prevalence (%)	Incidence (%)	Prevalence (%)	Incidence (%)	Prevalence (%)
Multimorbidity	6.68	29.87	1.65	3.11	6.40	13.09	8.43	20.60
NCD								
Hypertension	6.73	26.54	2.41	5.83	8.47	11.15	8.48	38.32
Cerebrovascular diseases	5.79	15.86	1.12	2.00	5.92	11.80	8.99	26.95
Ischemic heart disease	5.08	16.32	0.87	1.75	5.38	10.09	7.68	27.67
Diabetes	3.79	14.32	2.17	3.98	5.25	5.33	4.07	18.33
Arthritis	3.50	11.99	3.39	7.52	4.86	4.55	3.47	13.03
Cancer	2.84	7.54	2.14	3.47	3.94	3.84	2.94	8.07
Hyperlipidemia	1.69	4.89	0.88	1.77	2.46	2.27	1.73	5.30
Liver disease	1.53	3.81	1.52	2.48	2.36	1.65	1.25	3.29
COPD	1.33	4.01	0.19	0.44	0.75	3.58	2.73	8.48
Peptic ulcer disease	0.97	2.53	1.14	1.96	1.45	1.03	0.78	2.17
Chronic kidney disease	0.65	1.95	0.68	1.25	0.78	0.99	0.75	2.38
Asthma	0.56	1.75	0.60	1.39	0.73	0.79	0.60	1.91

*NCD* noncommunicable disease, *COPD* chronic obstructive pulmonary disease.

Of the overall population, 39.84% did not suffer from any of the included NCDs; 45.26% had only one NCD, while 14.87% exhibited multimorbidity, with an associated incidence of 6.68%. The incidence and prevalence of multimorbidity are also visually represented in Fig. 2A. The incidence of multimorbidity in different provinces/municipalities/autonomous regions is shown in Fig. 2B.

The 3 most common multimorbidity combinations for patients with 2 conditions were HT and CbVD (14.67% of patients suffer from only 2 NCDs); HT and IHD (13.30%); and diabetes and HT (10.36%). The 3 most common multimorbidity combinations for patients with 3 conditions were HT, IHD and CbVD (12.76%); diabetes, HT and IHD (9.81%); and diabetes, HT and CbVD (8.49%). The multimorbidity combinations in the different age subgroups are shown in Table 2.

**Table 2:** Multimorbidity combinations in different age subgroups.

Multimorbidity	18–34		34–64		≥ 65		Total	
2 conditions	HT, arthritis	8.86%	Diabetes, HT	11.88%	HT, CbVD	18.13%	HT, CbVD	14.67%
	Diabetes, HT	7.26%	HT, CbVD	11.27%	HT, IHD	16.13%	HT, IHD	13.30%
	HT, CbVD	6.99%	HT, IHD	10.61%	Diabetes, HT	9.16%	Diabetes, HT	10.36%
3 conditions	Diabetes, HT, arthritis	5.83%	Diabetes, HT, IHD	9.43%	HT, IHD, CbVD	16.91%	HT, IHD, CbVD	12.76%
	HT, IHD, CbVD	5.33%	Diabetes, HT, CbVD	7.50%	Diabetes, HT, IHD	10.24%	Diabetes, HT, IHD	9.81%
	Diabetes, HT, IHD	4.27%	HT, IHD, CbVD	6.94%	Diabetes, HT, CbVD	9.32%	Diabetes, HT, CbVD	8.49%
≥ 4 conditions	HLD, HT, IHD, arthritis	6.68%	Diabetes, HT, IHD, CbVD	7.37%	Diabetes, HT, IHD, CbVD	13.57%	Diabetes, HT, IHD, CbVD	11.16%
	Diabetes, HT, IHD, CbVD	5.30%	HLD, HT, IHD, Arthritis	5.96%	Diabetes, IHD, CbVD, Arthritis	8.73%	Diabetes, IHD, CbVD, Arthritis	6.89%
	Diabetes, HT, IHD, arthritis	5.11%	Diabetes, HLD, HT, IHD	5.82%	HLD, HT, IHD, CbVD	5.57%	HLD, HT, IHD, CbVD	5.45%

*NCDs* noncommunicable diseases, *HT* hypertension, *CbVD* cerebrovascular diseases, *IHD* ischemic heart disease, *HLD* hyperlipidemia.

For patients with at least one type of NCD, we calculated the RR of being in a multimorbidity condition for a single type of NCD compared to having other types of NCD to explore which type of NCD is more prone to coexist in a multimorbidity condition. The results of the calculations are shown in Fig. 2C. Of the 12 included NCDs, patients with HT had the highest RR for being in a multimorbidity condition (RR = 6.118, 95% CI 6.079–6.158), followed by IHD (RR = 5.237, 95% CI 5.205–5.270) and HLD (RR = 3.433, 95% CI 3.418–3.448). The lowest risk of multimorbidity among the 12 NCDs included was cancer (RR = 1.819, 95% CI 1.805–1.834).

**Figure 3:**
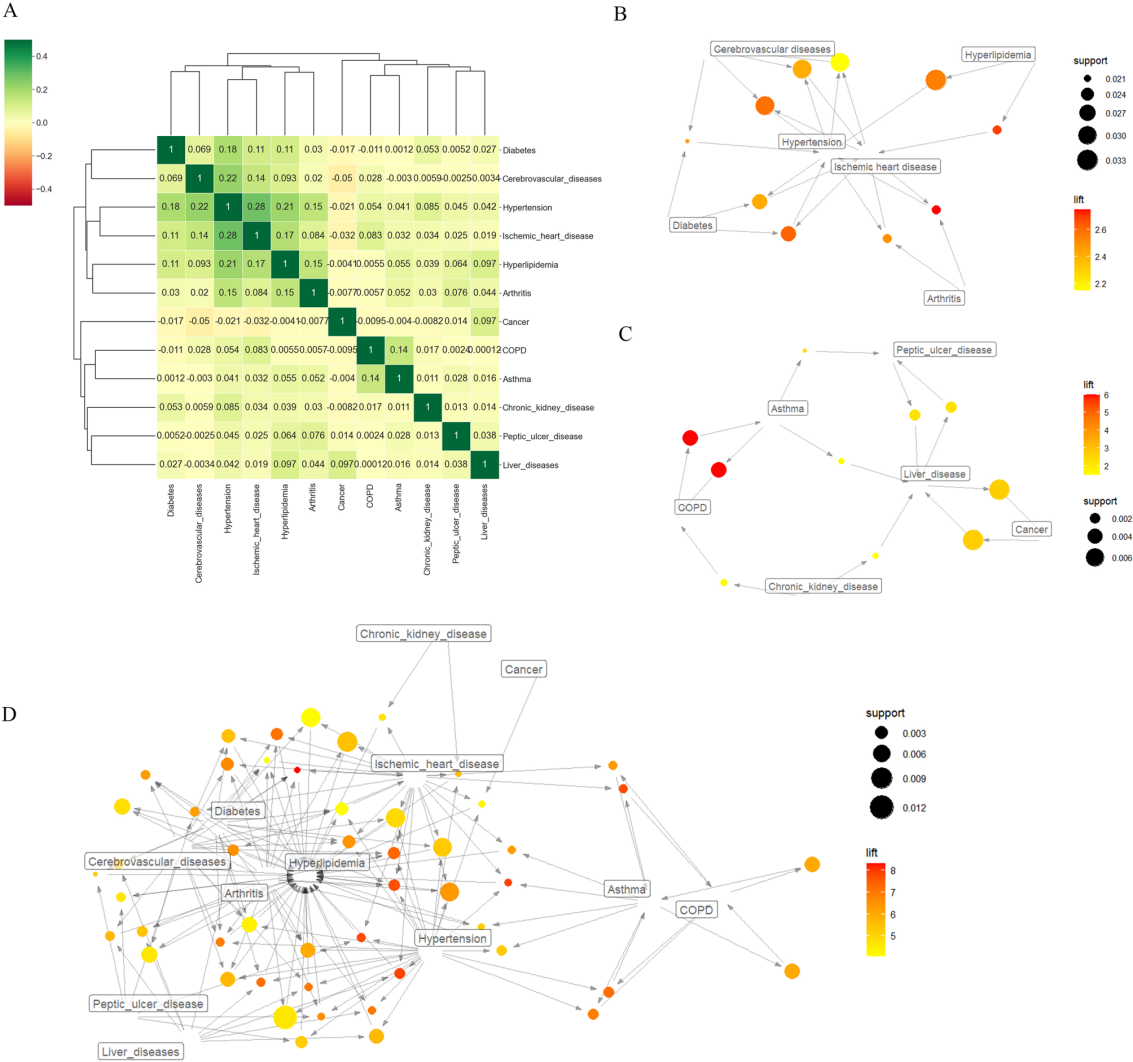
Analysis of multimorbidity patterns. (**A**) Cluster analysis results and correlation heatmap for multimorbidity. (**B**–**D**) Relationship diagram of cluster 1, cluster 2 and overall NCDs. The arrows represent the direction of the association rules. *COPD* chronic obstructive pulmonary disease.

Cluster analysis allowed 12 NCDs to be grouped into 2 different clusters (Fig. 3A) based on the diagnostic records of 955,354 patients. Cluster 1 included diabetes, asthma, HLD, HT, IHD and cerebrovascular diseases (CbVD). Cluster 2 included cancer, COPD, arthritis, peptic ulcer disease (PUD) chronic kidney disease (CKD) and liver disease (LD). A Pearson correlation matrix of approximations was used to describe the similarity of each NCD onset status, with the top five similarities ranked in order: HT and IHD, similarity of 0.28; HT and CbVD, similarity of 0.22; HT and HLD, similarity of 0.21; HT and diabetes, similarity of 0.18; IHD and HLD, similarity of 0.17.

Apriori ARM was applied to the 2 clusters generated by the clustering analysis. The NCDs in Cluster 1 produced 16 association rules (minimum support = 0.02, minimum confidence = 0.3), and the detailed results for the top 10 association rules with the highest lift are shown in Table 3. Ischemic heart disease and arthritis have the highest lift (lift = 2.682), indicating a high correlation between these two NCDs. In addition, IHD and HT have high lift along with high importance (support = 0.090) and reliability (confidence = 0.549). A visual relationship diagram of the results of Cluster 1 ARM is shown in Fig. 3B.

**Table 3:** Association rules calculated from the 2 NCD clusters.

Cluster	Antecedent	Consequent	Support	Confidence	Lift
Cluster 1	Hyperlipidemia	Ischemic heart disease	0.021	0.438	2.682
	Diabetes, ischemic heart disease	Hypertension	0.026	0.695	2.617
	Hyperlipidemia	Hypertension	0.033	0.676	2.548
	Diabetes, cerebrovascular diseases	Hypertension	0.021	0.654	2.463
	Diabetes, hypertension	Ischemic heart disease	0.026	0.395	2.418
	Ischemic heart disease	Hypertension	0.090	0.549	2.069
	Hypertension	Ischemic heart disease	0.090	0.338	2.069
	Diabetes, hypertension	Cerebrovascular diseases	0.021	0.309	1.950
	Cerebrovascular diseases	Hypertension	0.078	0.490	1.845
	Diabetes	Hypertension	0.067	0.465	1.752
Cluster 2	Cancer	Liver disease	0.008	0.103	2.703
	Liver disease	Cancer	0.008	0.204	2.703
	Peptic ulcer disease	Arthritis	0.007	0.273	2.274
	Arthritis	Peptic ulcer disease	0.007	0.058	2.274
	Liver disease	Arthritis	0.007	0.191	1.593
	Arthritis	Liver disease	0.007	0.061	1.593
	Arthritis	COPD	0.005	0.043	1.076
	COPD	Arthritis	0.005	0.129	1.076
	Arthritis	Cancer	0.008	0.070	0.927
	Cancer	Arthritis	0.008	0.111	0.927

*NCD* noncommunicable disease, *COPD* chronic obstructive pulmonary disease.

No strong association rules were obtained for the frequent item set in Cluster 2, and 10 association rules were obtained after lowering the parameters (minimum support = 0.005, minimum confidence = 0.01). Cancer and LD had the highest lift (lift = 2.703) but lower importance (support = 0.008) and reliability (confidence = 0.103). The specific results for Cluster 2 ARM are shown in Table 3, and the visual relationship diagram of the results for ARM is shown in Fig. 3C.

For the same reasons mentioned above, when the association rules are sorted by support value and the top 10 rules are retained, the ARM results on the entire dataset are identical to those of Cluster 1. Therefore, we reduced the parameter standards (support = 0.014, confidence = 0.05) so that each type of NCD was covered by at least one association rule when sorted in descending order of Support. Using this approach, we obtained 52 association rules, and the final selection included the top 50 rules for drawing the relationship diagram (Fig. 3D). The detailed results of the association rules for the 12 types of NCDs can be found in Supplementary Material S1.

In the overall population, the average number of annual outpatient visits was 12.6, and the average annual outpatient cost was $810 (with an average out-of-pocket ratio of 54.15%). The annual costs for patients with multimorbidity are approximately 2.4 times higher than those for patients without multimorbidity ($11,543 vs. $4863). The average annual cost of a single NCD is higher for cancer ($20,030 per year) and CKD ($9553 per year), which is much higher than the average cost of a single NCD ($6880 per year). In addition to multimorbidity, including cancer, the five multimorbidity combinations with the highest average annual costs are diabetes, HT and LD; diabetes, HLD and COPD; diabetes, HT, LD and HLD; diabetes, HLD and LD; and diabetes, HLD, COPD and HT. The specific costs for a single NCD and the 5 multimorbidity combinations with the highest costs are shown in Table 4.

**Table 4:** Costs for the 12 included NCDs and the five highest cost multimorbidity combinations.

Average annual costs		Outpatient	Inpatient	Total
NCD	Cancer	$3,066	$16,965	$20,031
	Chronic kidney disease	$4,749	$9,553	$14,302
	Arthritis	$618	$7,592	$8,210
	Hypertension	$757	$7,339	$8,096
	Hyperlipidemia	$628	$6,975	$7,603
	Liver disease	$709	$6,169	$6,878

*NCD* noncommunicable disease, *COPD* chronic obstructive pulmonary disease.

### Age-stratified analysis of NCDs and multimorbidity

**Figure 4:**
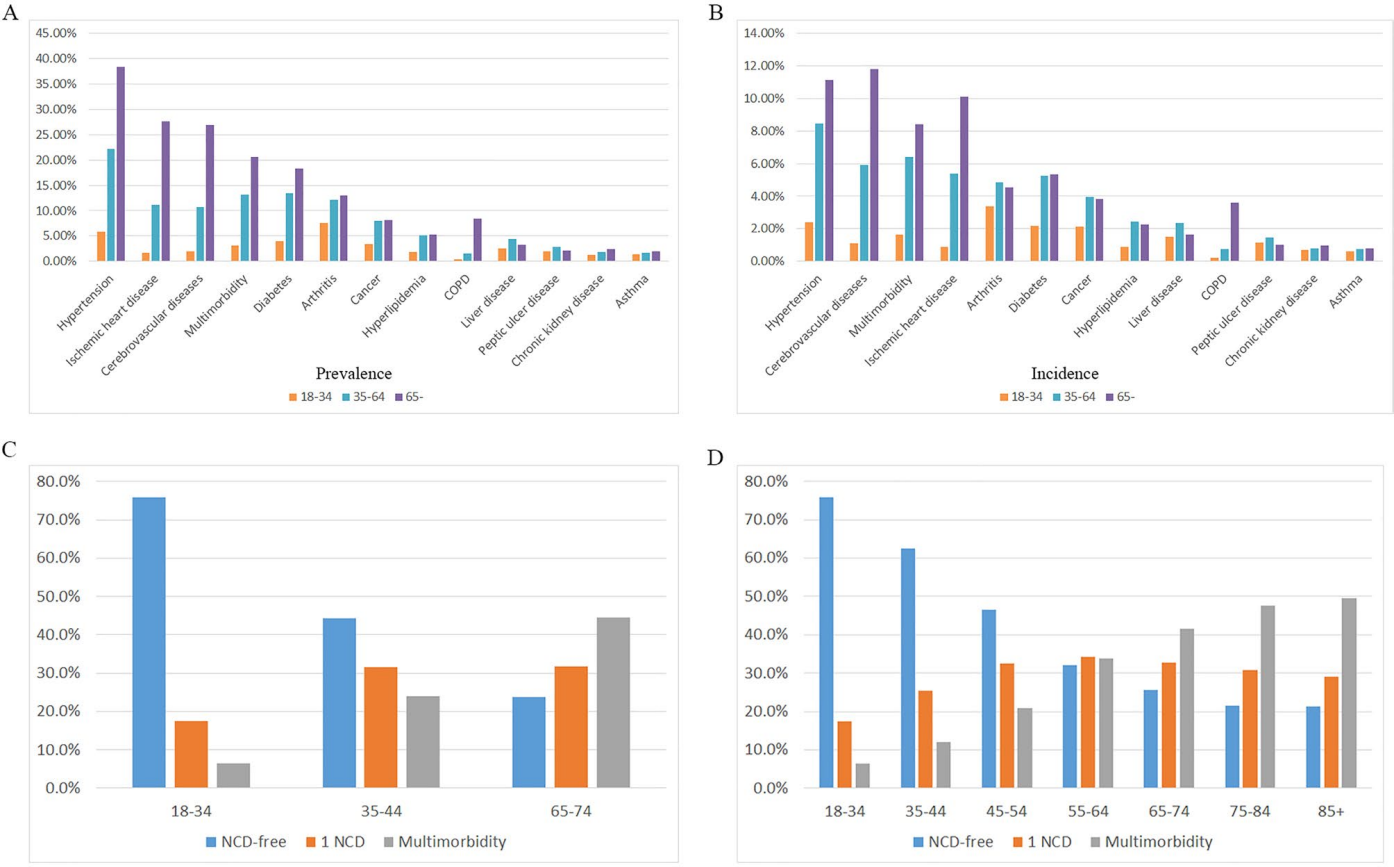
Subgroup analysis. (**A**) The incidence and (**B**) prevalence of the 12 included NCDs in individuals across three age subgroups. Multimorbidity proportions in three age categories (**C**) and five age categories (**D**).

In our subgroup analysis, individuals were stratified into three age groups. Individuals aged 18–34 were categorized as the "young group," those aged 35–64 as the "middle-aged group," and those aged 65 and older as the "elderly group." In the subgroup comprising individuals aged 18–34, the three most prevalent NCDs were arthritis, HT and diabetes. In the subgroup of individuals aged 35–64, the three most prevalent NCDs are HT, diabetes and arthritis. In the subgroup of individuals aged 65 years and older, the three most prevalent NCDs remained consistent with those observed in the overall population: HT, IHD and CbVD. In the young subgroup aged 35–64 years, the prevalence of multimorbidity was 3.11%, and the incidence was 1.65%. In the middle-aged subgroup aged 35–64 years, the prevalence of multimorbidity was 13.09%, and the incidence was 6.40%. In the older subgroup aged 65 and older, the prevalence of multimorbidity was 20.60%, and the incidence was 8.43%. The incidence and prevalence of the 12 included NCDs and multimorbidity in individuals across three age subgroups are depicted in Fig. 4A,B.

In patients identified as multimorbidity, young adults aged 18–34 accounted for only 2.18%, middle-aged individuals aged 35–64 comprised 42.68%, and elderly adults aged 65 and above constituted the largest proportion at 55.14%. The proportion of multimorbidity within different age subgroups is depicted in Fig. 4C,D. Binary logistic regression showed that the risk of multimorbidity increased with increasing age (p < 0.01). The risk of multimorbidity increased by 1.047 (95% CI 1.047–1.048) per 1-year increase in age. The comprehensive analysis results are presented in Table 5.

**Table 5:** Logistic regression analysis of multimorbidity presence by age.

		Variables in the equation							
		B	S.E	Wald	df	Sig	Exp(B)	Exp(B) 95%CI	
								Lower	Upper
Step 1^a^	Age	0.046	0.000	81,628.101	1	0.000	1.047	1.047	1.048
	Constant	-3.639	0.010	123,219.586	1	0.000	0.026		

Table 2 shows that the prevalent NCD combinations among the elderly population (aged 65 and above) are identical to those in the overall population, primarily characterized by co-occurrences of HT and cardiovascular/cerebrovascular diseases. The situation among the middle-aged group (34–64 years) closely resembles that of elderly individuals, but diabetes & HT constitute the highest proportion. Among the young adult group (18–34 years), the prevalence of multimorbidity contributed minimally to the overall multimorbidity rate, making it the most heterogeneous age group. The most frequent multimorbidity combination in this group was HT & arthritis.

**Figure 5:**
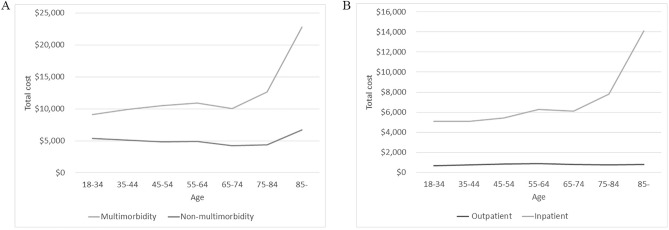
Average annual costs for different age subgroups. (**A**) Outpatient and inpatient costs in different age subgroups. (**B**) Multimorbidity and nonmultimorbidity costs in different age subgroups.

Among those aged ≥ 65 years, the average number of visits per year was 12.5, and the average annual outpatient cost was $792, which was slightly lower than the cost for those aged 35–64 years working ($844). In the overall population, the average number of annual outpatient admissions was 2.1, and the average annual cost of admissions was $6283. The overall trend in the number of admissions and cost of admissions increased with age. The total outpatient and inpatient costs in the different age subgroups are shown in Fig. 5A. The cost of multimorbidity was consistently higher than that of non-multimorbidity in all age subgroups, with this cost rising rapidly in the older age groups (Fig. 5B).

## Discussion

Previous studies on multimorbidity in China have often been limited in their representativeness. In our study design, we aimed to address these shortcomings. The UEBMI is the largest social medical insurance plan in China in terms of fund revenue and surplus^33^, accounting for 21.0% of the national health care system composition in 2013, according to the 2017 China National Health Statistics Yearbook. Our study was conducted by extracting desensitized data from UEBMI sources within the NICER database, and a sample of 1 million people was drawn from the attendance information of approximately 295 million urban population through a stratified sampling method, which is a good representation of the national urban population's attendance.

### Definition of multimorbidity and its incidence and prevalence

An important cause of bias in multimorbidity studies is the selection of predefined diseases. Multimorbidity studies are often conducted using predefined diseases, a consideration that is not only limited by objective conditions such as the methods of data collection but also economical in the statistical calculation of large-scale data. Different predefined disease categories and numbers can lead to bias in multimorbidity studies. In a review of multimorbidity studies, it was noted that the more diseases included for consideration, the higher the reported prevalence (Fortin et al.). They observed less variation of outcomes in studies that considered 12 or more diagnoses and therefore concluded that at least 12 diseases should be defined^17^. Our study adopted this recommendation, and due to our large sample size (1 million people sampled from 295 million), each additional disease put exponential upward pressure on the final statistical calculations, so we ended up selecting the 12 NCDs with high prevalence.

In the map of multimorbidity incidence (Fig. 2B), we noticed that Tianjin had a very high incidence of multimorbidity compared to the remaining country. We examined the underlying data and found that Tianjin had a high prevalence of IHD (42%). It was also observed in other studies^34^. An additional reason for this may be the bias introduced by the high outpatient detection rate of IHD in Tianjin (outpatient IHD/inpatient IHD ratio of 5.3 compared to the national average of 0.82). Trends in incidence rates were not significant as some regions were excluded from this study. In general, the incidence was higher in the South East.

### Age subgroups in association with multimorbidity

The most significant risk factor for multimorbidity is age, a finding that is very clear from previous studies of multimorbidity^35^. Figure 5C,D provide clear evidence of this trend, with NCD-free individuals being inversely related to age and individuals with multimorbidity showing a direct proportion to age. Nonetheless, limited research suggests a reduction in multimorbidity prevalence among elderly individuals, potentially linked to a bias arising from premature mortality stemming from the compounded health burden associated with multimorbidity^36–38^.

Our results for the incidence and prevalence of common NCDs in Chinese adults were similar to those of previous studies^39,40^, with the top 3 prevalent NCDs being HT, IHD and CbVD. The order of incidence in the elderly subgroup was approximately the same as that in the overall sample, but the highest incidence in the elderly subgroup was for IHD. Another notable point was that the incidence of COPD was significantly higher in the elderly subgroup than in young and middle-aged adults because age is one of the most important risk factors for the development of COPD. Nonetheless, the diagnosis of COPD is related to the disease state itself, the variability of diagnostic criteria, the difficulty of measuring COPD in the population and potential diagnostic bias, as a fixed FEV1/FVC ratio decreases with age in healthy individuals, and the widely used postbronchodilator spirometry-based diagnostic approach may lead to significant overdiagnosis in people over 50 years of age^41,42^.

Previous studies have shown that in analyses of age subgroups examining the burden of health care spending, spending generally increases with age^43–45^. However, our results showed that patients in the 35–64 age group spent slightly more on average per year in outpatient clinics than older people aged ≥ 65 years, which may be due to increased health literacy and health concerns among the younger age group in recent years. However, the average annual cost of hospitalization was much higher (30.44%) for the elderly population ($7436) than for the prime age group ($5701), and hospital expenditure was a major component of total health care expenditure. Our study found that multimorbidity led to significantly higher health care expenditures, which is consistent with previous studies^46^. In some countries in Europe and the US, multimorbidity accounts for more than half of healthcare expenditures, and the duration of illness increases with the life expectancy of the population^47^.

### Multimorbidity patterns

Commonly used methods for multimorbidity pattern studies are observed-to-expected ratio, cluster analyses, factor analyses, multiple correlation analyses, OR, and ARM^18^. We used a 2-step approach developed by Zemedikun et al.^32^, where 3 disease clusters were first found by cluster analysis and then an ARM was performed in the cluster containing more diseases to explore the characteristics between diseases in the cluster. In this study, the 12 included NCDs were divided into 2 clusters by cluster analysis, after which the ARMs were conducted for the 2 clusters separately.

The NCDs included in cluster 1 share a similar composition with cardiovascular and metabolic diseases^18,22^ or metabolic syndrome mentioned in previous studies, and we consider them to describe the same concept that has been widely noted. Furthermore, arthritis is classified into cluster 1, sharing a subcluster with HLD. Previous studies have reported an increased prevalence of metabolic syndrome and cardiovascular-related diseases in arthritis patients, advocating more frequent lipid profile assessments for these individuals^48,49^. However, this association was not substantiated by robust support in the ARM analysis between arthritis and HLD, as depicted in Fig. 3B. Instead, a weaker association was primarily observed with IHD. Therefore, our findings do not endorse the inclusion of HLD in the multimorbidity pattern of metabolic syndrome based on the aforementioned results. Rather, it underscores the importance of further investigating the relationship between arthritis and cardiovascular diseases.

Through the heatmap presented in Fig. 3A, it is evident that the six NCDs in cluster 2 can be classified into three distinct subclusters, with LD and PUD (gastrointestinal diseases) grouped together with CKD, suggesting a potential association between these conditions. Renal failure patients, owing to a high prevalence of 'functional' symptoms and medication-related factors, are more susceptible to the development of gastrointestinal and associated complications, including upper gastrointestinal lesions, acute and chronic gastrointestinal bleeding, pancreatitis, and ischemic colitis^50^. Asthma and COPD are classified as one cluster, i.e., respiratory diseases. Notably asthma-COPD overlap syndrome represents an important clinical phenotype that is more likely to present with respiratory symptoms and physical impairment and to report hospital admissions compared to asthma or COPD alone^51^. Cancer is a separate category, which may suggest that the occurrence of the 2 NCDs is more independent in cluster 2.

The ARM results for the six NCDs in cluster 1 show that HT and IHD were at the center of this association rule. Of the 10 association rules extracted from cluster 1, the strongest association was found between HLD and IHD (lift = 2.682), indicating that the probability of having IHD was 2.7 times higher in the presence of HLD than in the absence of HLD. HLD is a risk factor for IHD, the mechanism of which leads to the development of further IHD in patients with HLD because of the direct effect of lipid concentrations on the development of endothelial dysfunction^52^. The next strongest associations were for diabetes, IHD and HT (lift = 2.617), but the combination of these three diseases was not grouped in the same subcluster in the cluster analysis. The third strongest association was with HLD and HT (lift = 2.548). In cluster 2, the strongest association was found between cancer and LD (lift = 2.703), probably due to the high correlation between cirrhosis and liver cancer, with a significant proportion of cirrhosis being caused by liver cancer^53^. The importance and reliability of the association rules in cluster 2 are low, and we will not discuss the remaining rules further.

In addition, in the ARM conducted for all 12 NCDs in Fig. 3D, it is evident that HLD occupies a central position within the entire network of association rules. Among the 50 association rules used for constructing the network, HLD is observed a total of 13 times. Of these, 7 instances are located in the antecedent, implying that HLD may be one of the risk factors for other NCDs, making individuals with HLD more susceptible to other NCDs. The remaining 6 instances are situated in the consequent, which suggests the possibility that certain NCDs themselves may lead to the development of HLD or that the treatment of certain NCDs could result in HLD. It is important to note that ARM does not establish causality. Through ARM, we can observe the associations between HLD and other NCDs, but it does not determine whether HLD causes other NCDs, or vice versa, or if there are underlying common risk factors. Further research and data analysis are required to validate the nature and causes of these relationships.

Of the 12 NCDs included, HT (RR = 6.118) and IHD (RR = 5.237) were also at higher risk of multimorbidity than other NCDs. In conclusion, we consider that HT and IHD are two core conditions that should be of interest in multimorbidity care. When patients are identified with HT and IHD during outpatient or inpatient screening, attention should be given to whether they have other potential NCD diagnoses that may be consistent with multimorbidity. Additionally, at initial screening for HT and IHD, attention should be given to the prevention of NCDs such as diabetes and HLD. that may have a high likelihood of multimorbidity with the two core conditions mentioned above.

Other common NCDs, such as mental illness and obesity, were not included in our predefined 12 diseases. The variety of mental illnesses is more complex, and the underdiagnosis rate is relatively high^54^. In China, social stigma associated with mental illness may have led to more underdiagnosis^55^, and they are not usually included in studies of multimorbidity^2^, so we excluded psychiatric disorders from our study. Obesity is an important part of a multimorbidity development and morbidity pattern that increases the risk of other chronic diseases and increases healthcare costs^56^. However, we had to abandon the diagnosis of obesity because it is rarely included in clinical diagnosis in China, and we did not have information on the height, weight or BMI of the patients in our data.

### Limitations

There are some limitations to this study. First, although we met the minimum number of defined diseases recommended in previous studies^17^, there were some NCDs that were considered important in multimorbidity that were not included. Second, we used a database of Medicare billing records, which contains many potentially underdeveloped records of outpatient diagnoses with lower prevalence estimates compared to those based on selfreports and general practitioner reports^17^, so we used five consecutive years of records to attempt to avoid missed diagnoses. In addition, the population covered by health insurance may be biased in reflecting the overall population, as previous studies have shown that health care utilization is higher among those with social health insurance, and this effect is greatest among UEBMI individuals, thus potentially resulting in higher disease detection rates and thus higher incidence and prevalence rates in our calculations than the national average^43^. Finally, it should also be emphasized that multimorbidity is highly variable across the different populations investigated^54^, and the results of the study may not be fully representative of the population beyond the scope of the study.

## Conclusions

Our study suggests that the overall prevalence of multimorbidity was 6.68%, and the overall prevalence was 14.87% in 2017. The prevalence of multimorbidity increases with age. The pattern of the geographic distribution of multimorbidity shows that the prevalence of multimorbidity is relatively high in South East China. The average annual health care expenditure of patients with multimorbidity increased with age and rose rapidly, especially among older patients. Patients with cancer and CKD have higher treatment costs. Patients with HT or IHD had a significantly higher relative risk of multimorbidity than other included NCDs. HLD has generated the highest number of association rules, which may suggest that HLD may be both a risk factor for other NCDs and an outcome of them.

A more comprehensive understanding of the prevalence of multimorbidity in China will help healthcare workers and policy decision makers improve their understanding and knowledge of multimorbidity and achieve better expectations of health management for individuals, especially for older individuals (Supplementary Table S1).

### Supplementary Information

Supplementary Information 1.

Supplementary Table 1.

## Data Availability

The data that support the findings of this study are available from Beijing Healthcom Data Technology Co. Ltd but restrictions apply to the availability of these data, which were used under license for the current study, and so are not publicly available. Data are however available from the authors upon reasonable request and with permission of Beijing Healthcom Data Technology Co. Ltd.
