# Supplementary material for: Incidence, prevalence and characteristics of multimorbidity in different age groups among urban hospitalized patients in China
Source: Sci Rep. 2023 Nov 1;13:18798. doi: 10.1038/s41598-023-46227-4 (PMC10620234; doi:10.1038/s41598-023-46227-4)
Supplement: Supplementary file 1 — Supplementary Information 1. [file 41598_2023_46227_MOESM1_ESM.pdf]

Manuscript title: Incidence, prevalence and characteristics of multimorbidity in different age groups among urban hospitalized patients in China

Author list: Dixiang Song, Deshan Liu, Weihai Ning, Yujia Chen, Jingjing Yang, Chao Zhao, Hongwei Zhang

Supplementary material 1 Diagnosis entries and their corresponding ICD-10 codes used for auxiliary localization.

NCDs	Corresponding ICD-10 codes
Cancer	C00-C99, D00-D09
Diabetes	E10-E14, P70.0, P70.1, P70.2
Hyperlipidemia	E78.0-E78.5
Hypertension	I10-I15
Ischemic heart disease	I25
Cerebrovascular diseases	I60, I61, I63, I64, G45
COPD	J44.0, J44.1, J44.9
Asthma	J45
Peptic ulcer disease	K25-K28
Liver disease	K70-K77
Arthritis	M00-M25
Chronic kidney disease	N03, N11, N18

Please note: As mentioned in the original methodology section, ICD-10 is solely used as an auxiliary diagnostic localization method, and this table serves only as a means of disclosing the methodology.
