# Supplementary material for: Incidence, prevalence and characteristics of multimorbidity in different age groups among urban hospitalized patients in China
Source: Sci Rep. 2023 Nov 1;13:18798. doi: 10.1038/s41598-023-46227-4 (PMC10620234; doi:10.1038/s41598-023-46227-4)
Supplement: Supplementary file 2 — Supplementary Table 1. [file 41598_2023_46227_MOESM2_ESM.docx]

**Supplemental Table**

**S1 Association rules calculated from the overall 12 NCDs**

| Antecedent | Consequent | Support | Confidence | Lift |
| --- | --- | --- | --- | --- |
| Ischemic heart disease | Hypertension | 0.09 | 0.55 | 2.07 |
| Hypertension | Ischemic heart disease | 0.09 | 0.34 | 2.07 |
| Hypertension | Cerebrovascular diseases | 0.08 | 0.29 | 1.84 |
| Cerebrovascular diseases | Hypertension | 0.08 | 0.49 | 1.84 |
| Diabetes | Hypertension | 0.07 | 0.47 | 1.75 |
| Hypertension | Diabetes | 0.07 | 0.25 | 1.75 |
| Arthritis | Hypertension | 0.05 | 0.44 | 1.67 |
| Hypertension | Arthritis | 0.05 | 0.20 | 1.67 |
| Cerebrovascular diseases | Ischemic heart disease | 0.04 | 0.28 | 1.72 |
| Ischemic heart disease | Cerebrovascular diseases | 0.04 | 0.27 | 1.72 |
| Diabetes | Ischemic heart disease | 0.04 | 0.26 | 1.62 |
| Ischemic heart disease | Diabetes | 0.04 | 0.23 | 1.62 |
| Hyperlipidemia | Hypertension | 0.03 | 0.68 | 2.55 |
| Hypertension | Hyperlipidemia | 0.03 | 0.12 | 2.55 |
| Diabetes | Cerebrovascular diseases | 0.03 | 0.22 | 1.39 |
| Cerebrovascular diseases | Diabetes | 0.03 | 0.20 | 1.39 |
| Ischemic heart disease, Cerebrovascular diseases | Hypertension | 0.03 | 0.69 | 2.58 |
| Hypertension, Cerebrovascular diseases | Ischemic heart disease | 0.03 | 0.39 | 2.41 |
| Hypertension, Ischemic heart disease | Cerebrovascular diseases | 0.03 | 0.34 | 2.15 |
| Arthritis | Ischemic heart disease | 0.03 | 0.25 | 1.52 |
| Ischemic heart disease | Arthritis | 0.03 | 0.18 | 1.52 |
| Diabetes, Ischemic heart disease | Hypertension | 0.03 | 0.69 | 2.62 |
| Diabetes, Hypertension | Ischemic heart disease | 0.03 | 0.39 | 2.42 |
| Hypertension, Ischemic heart disease | Diabetes | 0.03 | 0.29 | 2.05 |
| Ischemic heart disease, Arthritis | Hypertension | 0.02 | 0.73 | 2.75 |
| Hypertension, Arthritis | Ischemic heart disease | 0.02 | 0.41 | 2.49 |
| Hypertension, Ischemic heart disease | Arthritis | 0.02 | 0.24 | 2.01 |
| Hyperlipidemia | Ischemic heart disease | 0.02 | 0.44 | 2.68 |
| Ischemic heart disease | Hyperlipidemia | 0.02 | 0.13 | 2.68 |
| Arthritis | Cerebrovascular diseases | 0.02 | 0.18 | 1.12 |
| Cerebrovascular diseases | Arthritis | 0.02 | 0.13 | 1.12 |
| Arthritis | Diabetes | 0.02 | 0.17 | 1.20 |
| Diabetes | Arthritis | 0.02 | 0.14 | 1.20 |
| Diabetes, Cerebrovascular diseases | Hypertension | 0.02 | 0.65 | 2.46 |
| Diabetes, Hypertension | Cerebrovascular diseases | 0.02 | 0.31 | 1.95 |
| Hypertension, Cerebrovascular diseases | Diabetes | 0.02 | 0.27 | 1.85 |
| Hypertension, Ischemic heart disease | Hyperlipidemia | 0.02 | 0.20 | 4.05 |
| Hyperlipidemia, Hypertension | Ischemic heart disease | 0.02 | 0.54 | 3.29 |
| Hyperlipidemia, Ischemic heart disease | Hypertension | 0.02 | 0.83 | 3.12 |
| Cancer | Hypertension | 0.02 | 0.23 | 0.88 |
| Hypertension | Cancer | 0.02 | 0.07 | 0.88 |
| Hyperlipidemia | Arthritis | 0.02 | 0.33 | 2.76 |
| Arthritis | Hyperlipidemia | 0.02 | 0.14 | 2.76 |
| COPD | Hypertension | 0.02 | 0.38 | 1.44 |
| Hypertension | COPD | 0.02 | 0.06 | 1.44 |
| Hyperlipidemia | Diabetes | 0.02 | 0.31 | 2.15 |
| Diabetes | Hyperlipidemia | 0.02 | 0.11 | 2.15 |
| Hyperlipidemia | Cerebrovascular diseases | 0.02 | 0.31 | 1.94 |
| Cerebrovascular diseases | Hyperlipidemia | 0.02 | 0.10 | 1.94 |
| Cerebrovascular diseases, Arthritis | Hypertension | 0.01 | 0.68 | 2.58 |
| Hypertension, Arthritis | Cerebrovascular diseases | 0.01 | 0.28 | 1.74 |
| Hypertension, Cerebrovascular diseases | Arthritis | 0.01 | 0.19 | 1.57 |
